# Neural Correlates of Own Name and Own Face Detection in Autism Spectrum Disorder

**DOI:** 10.1371/journal.pone.0086020

**Published:** 2014-01-22

**Authors:** Hanna B. Cygan, Pawel Tacikowski, Pawel Ostaszewski, Izabela Chojnicka, Anna Nowicka

**Affiliations:** 1 Nencki Institute of Experimental Biology, Department of Neurophysiology, Laboratory of Psychophysiology, Warsaw, Poland; 2 Karolinska Institute, Department of Neuroscience, Brain, Body and Self Laboratory, Stockholm, Sweden; 3 University of Social Sciences and Humanities, Department of Psychology, Warsaw, Poland; 4 Medical University of Warsaw, Department of Medical Genetics, Warsaw, Poland; University Children's Hospital Tuebingen, Germany

## Abstract

Autism spectrum disorder (ASD) is a heterogeneous neurodevelopmental condition clinically characterized by social interaction and communication difficulties. To date, the majority of research efforts have focused on brain mechanisms underlying the deficits in interpersonal social cognition associated with ASD. Recent empirical and theoretical work has begun to reveal evidence for a reduced or even absent self-preference effect in patients with ASD. One may hypothesize that this is related to the impaired attentional processing of self-referential stimuli. The aim of our study was to test this hypothesis. We investigated the neural correlates of face and name detection in ASD. Four categories of face/name stimuli were used: own, close-other, famous, and unknown. Event-related potentials were recorded from 62 electrodes in 23 subjects with ASD and 23 matched control subjects. P100, N170, and P300 components were analyzed. The control group clearly showed a significant self-preference effect: higher P300 amplitude to the presentation of own face and own name than to the close-other, famous, and unknown categories, indicating preferential attentional engagement in processing of self-related information. In contrast, detection of both own and close-other's face and name in the ASD group was associated with enhanced P300, suggesting similar attention allocation for self and close-other related information. These findings suggest that attention allocation in the ASD group is modulated by the personal significance factor, and that the self-preference effect is absent if self is compared to close-other. These effects are similar for physical and non-physical aspects of the autistic self. In addition, lateralization of face and name processing is attenuated in ASD, suggesting atypical brain organization.

## Introduction

Autism spectrum disorder (ASD) is a heterogeneous neurodevelopmental disorder which affects, according to various sources, from 1 in 160 children (WHO: www.who.int) to 1 in 88 (CDC: www.cdc.gov). The main clinical hallmarks of ASD include impairments in social functioning and communication, existence of stereotyped repetitive behaviors, and a highly restricted scope of interests. The precise neuropathophysiology of ASD is still unclear [1], [2].

While previous research has been largely focused on deficits in interpersonal (social) interaction in ASD [3]–[6], the current approach emphasizes the need for also understanding alterations in intrapersonal (self-referential) cognition [7], [8]. This need seems to be fully justified, as the term ‘autism’ (derived from the Greek word ‘autos’, meaning ‘self’) was first applied by Kanner to describe young patients from his clinic who were extremely self-focused [9]. Recently, Lombardo and Baron-Cohen [10] proposed that individuals with ASD can be both egocentric and impaired in self-referential cognition. The authors also pointed to the atypical neural circuitry underlying the processing of self-relevant information in ASD. In addition, Rogers and Pennington [11] suggested that a disturbed process of forming and coordinating representations of the self and the other may be linked to specific deficits observed in ASD.

Despite distinct methodological approaches and operationalizations of self-concept, many studies on autistic self consistently point to a lack of differences between representations of self and other [8], [12]–[14]. For example, in Gunji's event-related potentials (ERP) study [12], children with pervasive developmental disorder (PDD; PDD includes ASD) were passively viewing their own, familiar, and unfamiliar faces. Their ERP response to own face did not differ from ERP responses to familiar and unfamiliar faces, whereas in typically developing participants ERPs were enhanced in the self face condition in comparison to the familiar face condition. Parallel effects were found by Lombardo et al. [8] in a study using functional magnetic resonance imaging (fMRI). They asked individuals with ASD and control participants to make reflective mentalizing or physical judgments about themselves and an “other” (the British Queen). In ASD participants, the self and other conditions resulted in similar ventromedial prefrontal cortex activations, and the middle cingulate cortex responded even stronger to other-mentalizing than self-mentalizing. In contrast, neurotypical individuals preferentially recruited those regions in response to self when compared to other referential processing. Reduced or even absent self-preference effects in ASD participants were also reported in studies on the self-reference effect in memory (i.e. enhanced memory for stimuli encoded in reference to oneself). In Henderson's et al. [13] study, participants read a list of words and decided whether the word described something about themselves, something about Harry Potter, or contained a certain number of letters. In the following session, subjects were asked to recognize previously presented words on a long list. Consistent with previous studies [7], [14], ASD subjects showed a reduced or absent self-reference effect.

The aforementioned studies revealed some significant alterations in self-related information processing in ASD individuals and in the associated neuronal circuitry. These alterations may be viewed in the light of the absent-self hypothesis [15]–[21] and the impaired I-concept hypothesis [22]. The absent-self hypothesis proposes that a specific kind of higher order self-awareness, possibly involved in top-down control, may be missing in autism. The second hypothesis posits that development of I-concept in patients with autism is disturbed or even absent. Specifically, Glezerman [22] pointed to the impairment of the ‘symbolic’ self that is developed in the neurotypical population through lifetime experience and enables the perception of self as unique and separate from others.

One may hypothesize, however, that explanations referring to attentional processes seem to be reasonable. The impaired self-preference observed in many ASD studies may be related to weaker engagement of attentional resources in processing of self-related information. It is well-documented that in a typically developing population, stimuli referring to one's own person attract attention automatically [23] and are selectively detected among other stimuli in the environment. A good example is the so called ‘cocktail party’ effect. Even when engaged in another cognitive task, a person can still detect own name in the unattended ear or visual field [24]–[27]. Moreover, one's own name is particularly resistant to attentional blink [28], and is preferentially processed even without reaching conscious awareness [29]. Studies on the neural processing of self-related cues (one's own name or face) generally support this notion of preferential attention allocation [30]–[36].

Thus the question arises whether attention allocation for self-related stimuli is also disturbed in ASD patients. In order to answer this question, the present study investigated detection of one's own face and one's own name in ASD participants and matching control subjects. We decided to use a simple detection task because such tasks do not require any intentional discrimination between presented stimuli, engage attention automatically, and require the same motor reaction (i.e., pressing the same button) for all stimuli. As a result we can imply that any plausible differences between stimuli, conditions, or groups can be related to different activation of attentional processes. It is important to note that detection is an obligatory initial stage in the processing of any incoming stimulus. One may suppose that any impairment present at this stage of information processing determines alterations at later stages.

Up to now, no studies have compared the processing of own face and own name in the same group of ASD participants while using the same experimental paradigm. Such a comparison would enable us to relate to Uddin's hypothesis [37], stating that ‘physical’ aspects of the autistic self are less disturbed than ‘psychological’ (i.e. non-physical) aspects. Whereas self-face directly refers to the ‘physical self’, self-name refers rather to the ‘non-physical self’. If there is an atypical pattern of attention allocation for all self-related stimuli in ASD, similar results for names and faces should be observed; otherwise, some alterations might be observed for one type of stimulus only.

It is noteworthy that little is known about neural processing of one's own name in ASD. This is quite surprising given that names are highly relevant stimuli in the context of communication, i.e., the domain which is clearly impaired in this clinical group. To the best of our knowledge, there is only one published study on the neural basis of the own name processing in ASD. Carmody et al. [38] compared neural correlates of processing of one's own name, numbers, and the word ‘Hello’ in one ASD patient. Own name was associated with activations in the right frontal medial and middle gyri. Interestingly, self-name processing typically results in increased medial prefrontal cortex activation in normal populations [39]–[42]. However, because it was a single case study and because the subject was sedated during scanning, the results need to be treated with caution. Thus the investigation of own-name processing in ASD is of interest *per se* and may be viewed as a novel contribution to the field of ASD research.

In our study, control conditions consisted of names and faces belonging to three categories: unfamiliar, famous, and related to significant other. The latter category was introduced because ‘me’ vs. ‘not-me’ distinction may be modulated by the level of familiarity of the person used in the self-other comparison; it might be stronger for distant (not personally known and significant) other and weaker for close-other. Such modulation was reported in our previous studies of the neurotypical populations [42], [43]. The name and face of the close-other share many features with own name and face: their emotional load is very high, they are very familiar, and they are encountered extremely often in every-day life. Thus attention allocation for self and close-other related information may be similar, and processing of information related to close-other may resemble processing of self-related information.

The goal of this ERP study is to investigate the neural correlates of name and face detection in ASD. Specifically, we aim to verify our hypothesis stating that attention allocation for self-related stimuli is disturbed in ASD patients. This hypothesis would be confirmed if the self-preference effect expected in the control group is absent in the ASD group. Moreover, we are interested in whether the attentional involvement in detection of stimuli related to the ‘physical’ self (i.e., the own face) differs from attentional engagement in detection of stimuli related to the ‘non-physical’ self (i.e., the own name). Showing that the processing of own face is less disturbed than processing of own name would be supportive of Uddin's hypothesis [37].

The ERP method was chosen because it provides insights into the neural mechanisms that underlie covert cognitive processing that may not be evident in overt behaviors. Therefore, this method is particularly helpful when there might be no difference in a measured behavior between groups despite the supposition that the underlying neural substrate of that behavior may be different [44]. Amplitudes and latencies of the following ERP components were analyzed: P100 (a positive component peaking approximately 100 ms after the stimulus onset), N170 (a negative deflection reaching its maximum 170 ms after the stimulus onset), and P300 (an ERP component starting around 300 ms after the stimulus onset).

P100 and N170 components reflect exogenous processes modulated by the physical attributes of stimuli but not by cognitive processes [45]. Herrmann and Knight [46] proposed that these components are related to attention processes, operating at the early stage and influencing stimulus processing at the later stage. Many studies have shown that P100 reflects a facilitation of early sensory processing of attended stimuli [47], [48] and it may serve as a marker of early stimulus-driven attention allocation [49], [50]. In addition, it has been also proposed that P100 component may serve as a sign of processing effort [51]. In other words, the higher P100 amplitude (and/or the longer latency) the stronger need for engagement of brain resources.

In typical adults, the N170 component is related to early stage encoding of faces [52], [53]. The N170 is maximal over posterior areas and is faster and larger in response to face stimuli compared to non-face stimuli [53]. N170 was shown to be sensitive to face inversion [52]. Some studies revealed that it is affected by face familiarity whereas other did not find such effect [52]–[55]. It is now generally acknowledged that N170 represents the analysis of structural information of faces [53], [56]–[59]. Importantly, N170 is also specific to other stimuli processing that required expertise and was associated with word form analysis in case of names [43], [60].

The P300 component, in turn, has been mainly associated with the processes of attention and is often treated as an index of ability to sustain attention on targets [61]. Attention allocation reflected in P300 is independent of stimulus modality and is influenced by the familiarity factor [34], [36], [43]. The P300 also seems to vary with the emotional value of the stimulus - emotionally charged stimuli (regardless of their valence) produced larger P300 then neutral ones [62], [63]. There is still much debate on the underlying generator(s) but the prevailing opinion is that multiple neural sources contribute to the P300 [64].

These ERP components (i.e., P100, N170, P300) were observed and analyzed in previous studies on processing of faces and names in typically developing population [31], [32], [36], [65]–[74]. However, in the case of adult individuals with ASD, studies on face processing (there is no ERP study on name processing in the ASD) reported findings mainly related to P100 and N170 components. It has been demonstrated that adults with autism had delayed P100 and N170 latencies and lower N170 amplitudes for faces [75]. Other studies confirmed longer N170 latency in response to face stimuli in individuals with ASD but no significant effects for P100 and N170 amplitude and P100 latency were identified [76], [77]. In the recent Webb et al. study [78] no group differences in early ERP correlates of attention (P100) and structural face processing (N170) were found, suggesting that the P100 and N170 responses to upright faces in adults with ASD can resemble those seen in controls.

The majority of ERP studies on the topic of own name and own face processing report the self-preference effect in amplitudes of P300 in typically developing population [31], [32], [36], [72]–[74] (but see [79]). While P100 and P300 are the main candidates to differentiate attentional processes involved in detection of names and faces in the ASD group and the control group, only the latter component is associated both with the self-preference effect and attention allocation. Therefore, we expect that amplitudes of P300 in the ASD group will reflect plausible impairment of attentional processes involved in the processing of self-related stimuli. Such impairment should be manifested as a lack of differences between P300 amplitudes in the self vs. other condition and may be influenced by the personal relevance of ‘the other’.

## Methods

### Ethics statement

The experimental protocol was approved by the Bioethics Committee of Warsaw Medical University (Warsaw, Poland). Informed written consent was obtained prior to the study from all participants and their legal caregivers.

### Participants

Twenty three adolescents and young adults with ASD and 23 control subjects participated in this study (age range 17–27 years). ASD subjects were recruited from the SYNAPSIS Foundation which provides diagnosis and therapy for people with ASD. The subjects' IQs were evaluated on the basis of the Wechsler Intelligence Scale for Adults - Revised (WAIS-R) Polish adaptation [80]. The control group was matched in terms of age, handedness, and IQ-score (see Table 1). ASD subjects were clinically diagnosed by psychiatrists prior to the experiment and the clinical diagnosis was confirmed using standardized tests: the Autism Diagnostic Observation Schedule (ADOS) and Autism Diagnostic Interview-Revised (ADI-R) (see Table 1). Handedness was confirmed with the Edinburgh Inventory [81]. Subjects had normal or corrected-to-normal vision. All subjects were financially compensated for their participation in the experiment.

**Table 1:** Participants' characteristics in the ASD group (age, handedness, IQ, ADOS, and ADI-R scores) and in the control group (age, handedness, and IQ scores).

				IQ			ADI-R			ADOS		
subject	group	age	handedness	verbal	performance	full scale	social	communication	repetetive behavior	social	communication	repetitive behavior
							(cutoff = 10)	(cutoff = 8)	(cutoff = 3)	(cutoff = 4)	(cutoff = 2)	
A1	ASD	18	L	113	112	113	25	20	6	11	4	2
A2	ASD	23	R	100	69	86	25	23	7	3	3	1
A3	ASD	23	R	108	122	114	30	26	12	9	6	0
A4	ASD	19	R	96	90	93	26	18	8	8	5	0
A5	ASD	19	R	109	103	106	27	26	12	6	3	0
A6	ASD	18	R	116	93	106	27	21	11	8	3	2
A7	ASD	17	R	104	118	112	26	14	5	11	6	2
A8	ASD	17	L	119	121	122	27	21	8	5	3	3
A9	ASD	22	R	119	122	121	25	17	5	9	3	3
A10	ASD	18	R	96	109	102	25	23	8	8	5	2
A11	ASD	19	R	108	83	97	21	22	8	6	6	7
A12	ASD	18	R	125	107	117	25	22	10	7	3	3
A13	ASD	19	R	114	123	118	24	17	9	12	4	1
A14	ASD	24	R	124	93	111	16	21	7	5	2	4
A15	ASD	19	R	85	95	89	30	20	12	13	4	1
A16	ASD	22	R	97	121	108	24	17	2	4	2	0
A17	ASD	27	R	98	121	108	23	42	11	3	3	0
A18	ASD	21	R	99	104	101	26	22	9	8	3	2
A19	ASD	24	R	143	107	128	8	20	5	5	5	2
A20	ASD	21	R	114	118	116	14	8	8	3	3	0
A21	ASD	18	R	123	110	118	14	16	11	10	4	0
A22	ASD	21	R	119	99	110	12	15	1	4	2	0
A23	ASD	23	R	112	101	108	24	18	4	8	4	2
mean		20,4		109	104	107,13						
(s.d.)		2,76		11,3	16,43	11,51						
C1	Control	18	L	110	110	110						
C2	Control	17	L	122	92	109						
C3	Control	19	R	130	97	116						
C4	Control	19	R	86	99	91						
C5	Control	18	R	120	112	117						
C6	Control	19	R	111	108	110						
C7	Control	22	R	116	126	121						
C8	Control	23	R	107	114	110						
C9	Control	18	R	130	123	128						
C10	Control	23	R	139	122	132						
C11	Control	18	R	86	93	89						
C12	Control	22	R	120	125	123						
C13	Control	23	R	99	93	97						
C14	Control	17	R	116	117	117						
C15	Control	19	R	114	112	113						
C16	Control	22	R	120	125	123						
C17	Control	27	R	113	113	113						
C18	Control	21	R	111	118	114						
C19	Control	24	R	117	116	117						
C20	Control	21	R	119	103	112						
C21	Control	24	R	113	101	108						
C22	Control	21	R	119	118	119						
C23	Control	23	R	120	121	121						
mean	Control	20,8		114	109,53	112,20						
(s.d.)	Control	2,66		15	12,13	12,36						

All subjects were male.

### Stimuli

Faces and names (first and last names) were presented visually on a computer screen in two separate sessions. The sequence of the two sessions was randomized between subjects: half started with the name-detection task, while the other half with the face-detection task.

In the face-detection session, grey-scaled images of faces were presented against a black background. All photos were extracted from the original background using Adobe Photoshop CS5® software (Adobe Systems Incorporated), so that only the face, ears, and hair were visible. Faces belonged to four categories: (1) subjects own, (2) close-other's, (3) famous person (e.g., actor), and (4) unknown face. A face from each category was presented 32 times. The photos of famous and unknown people were downloaded from the internet. The luminance of pictures was matched to color statistics of one image, eliminating possible differences between stimuli. The size of the face stimuli ranged from 6°×6° to 6°×5°, and did not differ between categories or groups.

Names were written in white, capital letters and presented against a black background. Categories of names were analogous to categories of faces: (1) subjects own, (2) close-other's, (3) famous person (e.g., actor), and (4) unknown name. A name from each category was presented 32 times. The size of the name stimuli ranged from 3°×6° to 3°×9° and did not differ between categories or groups. Stimuli in both series were presented in pseudo-random order, so that no more than three stimuli of the same category or type were presented consecutively.

The set of all stimuli was individually tailored. Different famous and unknown faces/names were chosen for each subject to match gender of faces and length of the own and close-other's names. Names and faces of analogous categories referred to the same person. Before the experiment each participant was asked to confirm that he knows the famous person and does not know the unknown names. No restriction was put on the subjects choice of the close-other because we wanted to avoid a situation where predefined the ‘close-other’ is not really close to the subject. In the ASD group 16 participants chose their parent, three their sibling, three their grandmother, and one their best friend. In the control group, seven participants chose their parent, four their sibling, three their best friend, and nine their girlfriend.

### Experimental procedure

Stimuli were displayed in central vision on a 19-inch NEC MultiSync LCD 1990Fx monitor. Presentation® software (Neurobehavioral Systems, Albany, CA, USA) was used for stimuli presentation and measurement of the subject responses. The participants were seated in an acoustically and electrically shielded dark room at a distance of 60 cm from the computer monitor. The subjects performed a simple detection task: they were to respond to each stimulus as quickly as possible by pressing the same button with their index finger on a Cedrus response pad (RB-830, San Pedro, USA).

After reading instructions displayed on the computer screen, each session began with the participant completing a trial session in which feedback information was displayed (i.e., “correct”, “response too slow”). During this session stimuli from each category were presented twice. After succesful completion, subjects began the actual study.

The sequence of events in each trial was as follows: presentation of a fixation point (a white “+” against a black background) for 100 ms, a blank screen for 300 to 1200 ms, and a target item displayed for 500 ms. Onset of the consecutive trial was driven by the subjects response and appeared 2000 ms after pressing the response button. Following the first session, the second one (preceded by the training session) was initiated by the subject by pressing a response button. Each session lasted about 7 minutes.

### EEG recordings

EEG was continuously recorded from 62 scalp sites using a 136-channel amplifier (QuickAmp, Brain Products, Enschede, the Netherlands) and BrainVisionRecorder® software (Brain Products, Munich, Germany). Ag-AgCl electrodes were mounted on an elastic cap (ActiCAP, Munich, Germany) and positioned according to the extended 10–20 system. Electrode impedance was kept below 5 kΩ. The EEG signal was recorded against an average of all channels calculated by the amplifier hardware. The sampling rate was 500 Hz.

### Behavioral data analysis

Responses were scored as correct if the button was pressed within 150–1000 ms after the stimulus onset. Response times (RTs) were analyzed using mixed-model ANOVA, with the following factors: group (ASD, control), type (face, name), and category (own, close-other, famous, and unknown). RTs were averaged across correct trials only.

### ERP analysis

Off-line analysis of the EEG signal was performed using BrainVisionAnalyzer® software (Brain Products, Gilching, Germany). The first step was the implementation of butterworth zero phase filters: high-pass – 0.1 Hz, 12 dB/oct; low-pass – 30 Hz, 12 dB/oct; and notch filter – 50 Hz. Next, we corrected ocular artifacts using Independent Component Analysis [82]. After the decomposition of each data set into maximally statistically independent components based on visual inspection of the component map [83], components representing eye blinks were rejected. Ocular-artifact-free EEG data were obtained by multiplying the remaining ICA components using the reduced component-mixing matrix. Then, the EEG signal was segmented to obtain epochs extending from 100 ms before to 1000 ms after the stimulus onset (baseline correction from −100 to 0 ms). In the automatic artifact rejection, the maximum permitted voltage step per sampling point was 50 µV. The maximum permitted absolute difference between two values in the segment was 200 µV. The minimum and maximum permitted amplitudes were −200 µV and 200 µV respectively, and the lowest permitted activity in the 100 ms interval was 0.5 µV. Finally, the data were re-referenced to the mean from both earlobes and averaged for each stimuli category.

The ERPs for own, close-other's, famous, and unknown faces/names were computed for correct trials only. The mean number of segments used to compute ERPs in the ASD group was 29 for names and 30 for faces. In the control group, 31 for names and 31 for faces. We did not find significant differences in the number of epochs used to compute ERPs between types and categories of stimuli or between groups.

In the statistical analysis, peak latencies and amplitudes were used. We analyzed amplitudes and latencies of P100, N170, and P300 previously reported in studies with visual presentation of faces and names, at previously reported locations [31], [32], [36], [65], [71]–[74], [79]. Based on the visual inspection of grand-average ERPs and on the existing literature, the peak detection was performed for the following time-windows: P100 (50–150 ms after the stimulus onset), N170 (150–220 ms), and P300 (250–450 ms). We focused on scalp regions in which those ERP components had their maximum amplitudes. P100 and N170 were analyzed in the left and right occipital regions (PO7 and PO8). P300 was analyzed in the central-parietal region (CPz, CP3 and CP4). Including two lateral electrodes (i.e., CP3, CP4) into the sub-set of centro-parietal electrodes enabled us to relate to the issue of plausible differences in the left and right hemisphere involvement in processing of names and faces in the ASD group. Our choice of electrodes was confirmed by the topography of brain activity in the time windows corresponding to P100, N170, and P300.

Taking into account that P100 and N170 were analyzed at the same electrode sites, amplitude of the second component was analyzed as a peak-to-peak against P100. Visual analysis of the P300 revealed double maxima within chosen time window. Thus peak detection was performed in two time windows: early P300 (250–350 ms) and late P300 (350–450 ms). Epochs were visually inspected to ensure that for each participant ERP components reached their maximum/minimum within the selected time window.

We performed mixed-model ANOVA on amplitudes and latencies of each component with the following factors: group (a between-subject factor at two levels: ASD, control), type (a within-subject factor at two levels: face, name), category (a within-subject factor at four levels: own, close-other, famous and unknown), and location. This within-subject factor was at two levels (left, right) in the case of P100 and N170 and at three levels (left, central, right) in the case of P300. All effects with more than one degree of freedom in the numerator were adjusted for violations of sphericity according to the Greenhouse and Geisser formula [84]. T-tests with Bonferroni correction for multiple comparisons were applied to post-hoc analyses. Only interactions involving the between-subjects factor of group that were necessary to address the main aims of the present study were further analyzed.

## Results

### Behavioral data

No significant main effects or interaction were found.

### Electrophysiological data

#### P100

**Figure 1:**
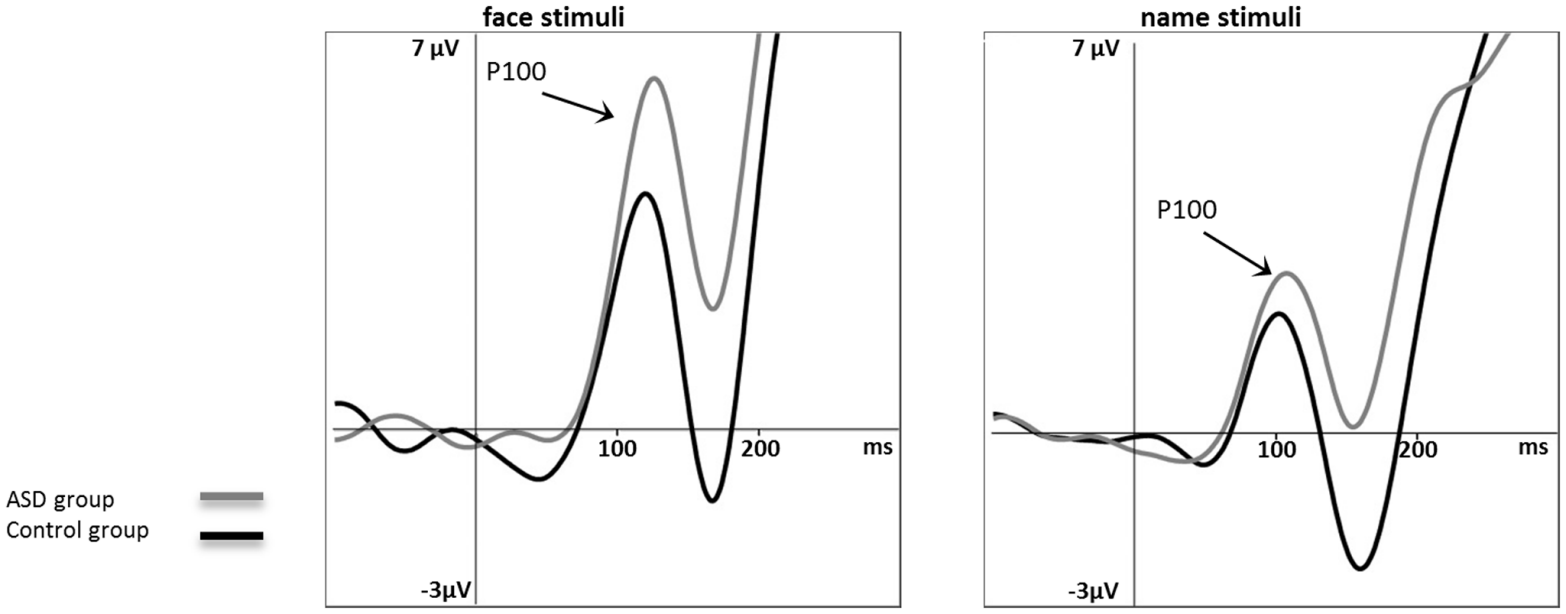
Grand average event-related potentials (ERP) in the P100 time window in the ASD group vs. the control group, pooled for PO7 and PO8 electrodes. left panel presents response to face stimuli, right panel presents response to name stimuli, with all categories taken together.

Statistical analysis on P100 amplitudes revealed a significant main effect of the type of stimulus (F_1,44_ = 20.968; p<.0001; η^2^ = .323) and two interactions: type × location (F_3,90_ = 4.971; p = .031; η^2^ = .102) and type × group (F_1,44_ = 3.916; p = .05; η^2^ = .820). All other main factors and interactions were insignificant. Faces, in general, were associated with higher P100 amplitudes than names. Post hoc tests showed that only face amplitudes were significantly higher in the right than in the left hemisphere (p = .019). Between-group differences referring to the type of stimulus showed that face amplitudes of P100 were higher in ASD than in the control group (p = .036) (see Figure 1). Analysis of the P100 latencies revealed a main effect of the type factor (F_1,44_ = 67.581; p<.0001; η^2^ = .606). Latencies for faces were significantly longer than for names.

#### N170

**Figure 2:**
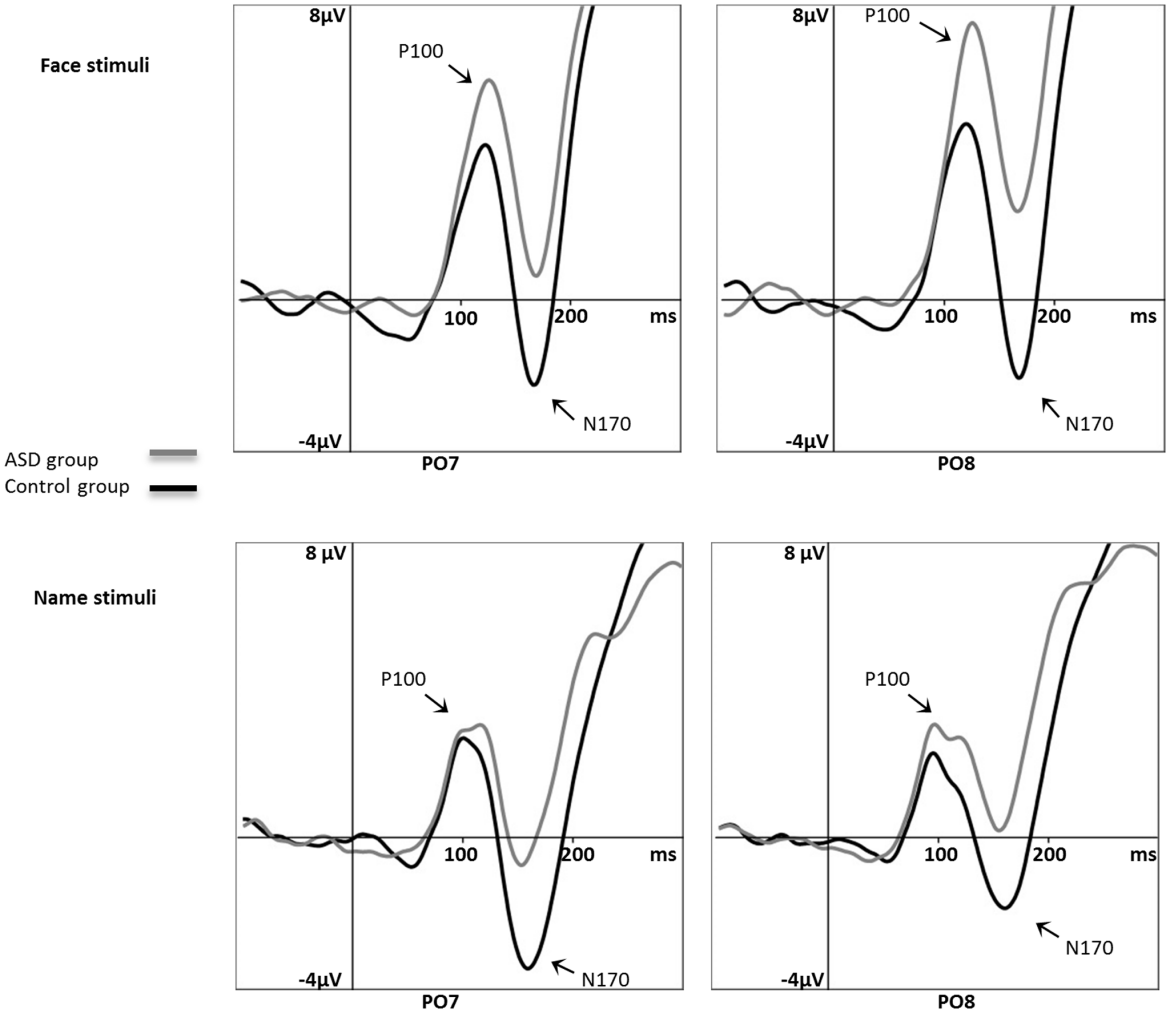
Grand average event-related potentials (ERP) in the N170 time window in the ASD group vs. the control group, separately for each analyzed scalp position (PO7, PO8). upper panels present response to face stimuli, bottom panels present response to name stimuli, with all categories taken together. For faces, amplitudes of N170 (peak-to-peak vs. P100) were higher on PO8 than on PO7 and for names amplitudes were higher on PO7 than on PO8.

Statistical analysis of N170 amplitudes revealed a main effect of category (F_1,44_ = 2.837; p = .04; η^2^ = .061) and a significant 3-way interaction: group × type × location (F_1,44_ = 5.138; p = .028; η^2^ = .105). Post hoc analysis showed that in the control group N170 amplitudes for names were higher in the left hemisphere than in the right (p = .014), and N170 amplitudes for faces were marginally higher in the right hemisphere than in the left (p = .085). Moreover, N170 amplitudes in the left hemisphere were higher in the control than in the ASD group (p = .004). Analysis of N170 latencies revealed a main effect of the type of stimuli (F_1,44_ = 12.954; p = .001; η^2^ = .227). N170 latency for faces was significantly longer than for names (see Figure 2).

#### P300

**Figure 3:**
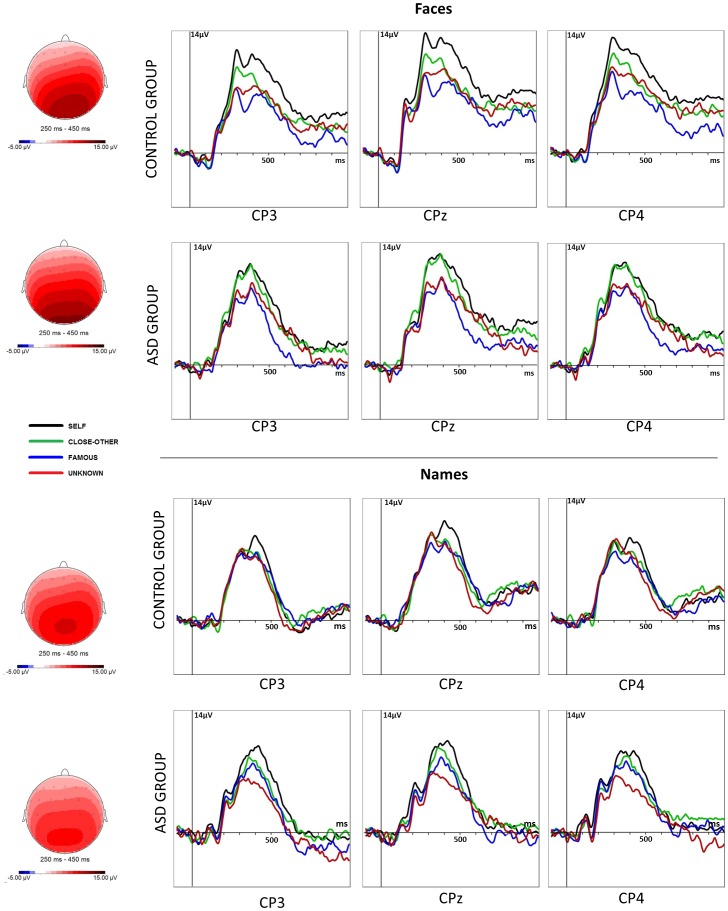
Grand average event-related potentials (ERP) in the ASD group vs. the control group, presented separately for each category of stimuli (own, close-other, famous, unknown), each analyzed centro-parietal scalp position (CP3, CPz, CP4), and each type of stimuli (face, name).

ANOVA for early P300 (see Figure 3) amplitudes revealed a main effect of the type of stimuli (F_1,44_ = 9.558; p = .003; η^2^ = 0.178), category of stimuli (F_3,42_ = 9.741; p<.001; η^2^ = .181), electrode location (F_2,43_ = 12.099; p<.001; η^2^ = .216), and interactions: type × category (F_3,42_ = 4.621; p = .004; η^2^ = .095), group × type × category (F_3,42_ = 3.997; p = .013; η^2^ = .083), group × type × location (F_2,43_ = 5.013; p = .009; η^2^ = .102), and group × category × location (F_6,39_ = 3.451; p = .005; η^2^ = .073). All other effects were insignificant. Post hoc tests of ‘group × type × category’ interaction showed that in both groups there were no differences in early P300 between categories of names. However, significant differences appeared in response to faces. In the ASD group, response to own face did not differ from the response to close-other's face and unknown face. However, amplitudes to own and close-other's face were higher than for famous face (p = .005 and p = .001, respectively). In the control group, response to own face was significantly higher than to the close-other's (p = .001), famous (p<.001), and unknown face (p = .001).

Post hoc tests of ‘group × type × location’ interaction showed that in the ASD group amplitudes for faces were higher than for names at each investigated location: left (p = .008), right (p = .007), and central (p = .002). In the control group, this effect appeared only on the right side of the scalp (p = .041). Post hoc analyses of the ‘group × category × location’ interaction revealed that in typically developing participants, amplitudes of early P300 to all categories of faces and names were higher at the right (CP4) and central (CPz) electrode sites in comparison to the left (CP3). In contrast, this effect (CPz, CP4>CP3) was observed in ASD subjects only for the close-other category.

Analysis of early P300 latencies revealed a main effect of the group (F_1,44_ = 4.776; p = .034; η^2^ = .970). Longer latencies of early P300 latencies were observed in the ASD group than in the control.

ANOVA on late P300 peak amplitudes (see Figure 3) revealed a main effects of the type of stimuli (F_1,44_ = 4.904; p = .032; η^2^ = .100), category of stimuli (F_3,42_ = 19.522; p<.001; η^2^ = .307), location (F_2,43_ = 8.978; p<.001; η^2^ = .169), and statistical significance of interactions: group × category (F_3,42_ = 3.243; p = .024; η^2^ = .068), category × location (F_6,39_ = 3.022; p = .013; η^2^ = .064), type × location (F_2,43_ = 3.168; p = .047; η^2^ = .067), and group × category × location (F_6,39_ = 3.699; p = .004; η^2^ = .078). All other main factors and interactions were insignificant. Post hoc tests of ‘group × category’ interaction indicated that in the ASD group, regardless of the type of stimuli, late P300 response to own face/name was higher than for famous (p = .002) and unknown face/name (p<.001). Peak amplitude for the close-other category was higher than for famous (p = .023) and unknown (p = .008). No differences between the self and close-other category were found. In the control group, response for the own face/name was higher than for the close-other's (p = .001), famous (p<.001), and unknown face/name (p = .001). In this group, peak amplitude for close-other's face/name was higher than for famous face/name (p = .014). Post hoc tests for the ‘group × category × location’ interaction showed that the described effects in each group were significant at each investigated electrode location. However, they were the strongest at the central site. No significant differences regarding P300 peak latencies were observed in the late time window.

## Discussion

The goal of this ERP study was to investigate the neural correlates of name and face detection in ASD. Names and faces differed in respect to their personal significance (own, close-other's, famous, unknown). Specifically, we were interested whether preferential attention allocation for self-related stimuli was impaired in ASD participants, and whether the same effects could be observed for the ‘physical self’ (one's own face) and the ‘non-physical self’ (one's own name).

On the behavioral level, we did not find differences in RTs between groups and experimental conditions. It should be stressed, however, that the task we used was very simple and did not require discriminating between stimuli. No in-depth processing of incoming information was required to successfully accomplish the task, and only high-functioning ASD participants were tested.

On the neural level, several significant effects were observed. In general, faces were associated with higher P100 amplitudes than names in both groups. It has been well documented that the amplitude of P100 is sensitive to perceptual features of visual stimuli, such as brightness, contrast, visual acuity, and size [85], [86]. Interestingly, some studies report face-sensitive effects at the level of P100 [73], [87]. It has been argued, however, that this may just result from perceptual differences between faces and visual stimuli used for comparison [88]. Thus increased P100 to faces in both our groups is possibly a consequence of the size and complexity of these stimuli. We observed no self-preference effect in this early ERP component for both the ASD and control group.

Besides the effects common for the two groups, some between-group differences appeared about 100 ms after the stimulus onset: P100 amplitudes in response to all categories of faces were higher in the ASD group than in the control group. This effect may reflect the enhanced visual processing often reported in ASD [89]. Alternatively, it could be also attributed to early stimulus-driven attention allocation [49], [50], i.e., enhanced P100 to faces in the ASD group may reflect increased orienting/attention to these stimuli. However, increased attention operating at the early stage of face processing did not exert influence on the later stages, related to face recognition (see discussion referring to the P300 findings below). Actually, one may speculate that this enhanced P100 indicates the higher processing effort present at earliest stage of face perception in this clinical group [51]. However, none of previously published studies on face processing reported P100 amplitudes to faces higher in individuals with ASD than in control individuals [75]–[78]. This discrepancy between our P100 results and findings of previously published studies may result from crucial differences in experimental paradigms, i.e. different attentional requirements, different stimuli and different subject's tasks. Specifically, in Webb et al. [78] and MacPartland et al. [77] studies, presentation of faces was task-irrelevant, thus faces were out of focus of attention: subjects were supposed to detect (i.e., press a button) houses [78] or butterflies [77]. In two other studies, emotional faces were presented [75], [76] and subjects were asked either to indicate whether the face was neutral or sad [76] or to verbalize the word which described how the person in the photograph was feeling [75].

Subsequently, amplitudes of N170 differentiated the two groups: N170 recorded in the left hemisphere to names was higher in the control group than in the ASD group. In addition, lateral effects related to the type of presented information were present only in the control group. Specifically, N170 amplitude to names was higher in the left hemisphere than in the right one and to faces higher in right than in the left one, however the second effect was only marginally significant. Although general enhancement of N170 amplitudes to faces is typically observed when faces are compared to other visual objects [90], some studies revealed higher amplitudes of N170 component in the right hemisphere only [43], [52], [91]. Increased N170 for names in the left hemisphere, in turn, was also previously reported in healthy subjects [73], [86], [92] and seems to be in line with typical dominance of the left side of the human brain in language processing.

While in the control group on the early stage of information processing, left hemisphere dominance appeared for visually presented names and right hemisphere dominance – for faces, such effects were absent in the ASD group. In the case of names, the lack of lateral effects is generally in line with the previously found dysfunction of the left hemisphere in the ASD [93], [94] (but see [95]) and atypical patterns of lateralization of language processing in this clinical group [96], [97]. In the case of faces, presence of the right hemispheric dominance in ASD groups is still unclear. For example lack of lateral differences in ASD was previously reported in N170 component by [98] while other studies report such lateralization for both control and ASD subjects [75], [76], [78].

With regard to the main aim of our study, the most important findings were between-group differences found in the 250–350 ms and 350–450 ms time windows. In both groups, P300 amplitudes in the early time window were significantly modulated by categories of faces, not names. In the control group, the own face was associated with higher P300 amplitudes than all other faces, whereas in the ASD group the own and close-other's face did not differ. Both resulted in enhanced P300 in comparison to the famous but not unknown face. The latter is in line with findings of a study reporting that children with autism fail to show differential late positive ERP component to their mother's face versus an unfamiliar face [99]. These higher amplitudes of early P300 to own and the close-other's face but not to the famous face seem to reflect the personal relevance of those stimuli. The lack of significant differences between personally relevant faces (i.e., own and close-other faces) and unknown faces possibly results from equivalent attention allocation for those stimuli. A similar effect in ASD children was found by Gunji et al. [12]. In this study, no significant differences in P300 components were observed among the own, familiar, and unfamiliar face conditions. We argue that this elevated level of attention may be due to an elementary adaptive mechanism that guarantees that events/information with a potentially high survival value would not be missed [100]. It might be the case that novel objects (unknown faces) attracted ASD participants' attention to the same extent as personally relevant but not famous faces. Latency of early P300 also differentiated the two groups. It was significantly longer in the ASD group than in the control group for all names and faces, indicating some delay in processing of these socially-relevant stimuli.

Importantly, in the late time window we observed common patterns of P300 amplitudes for both types of stimuli, indicating the self-preference effect in the control group and the personal relevance effect in the ASD group. Specifically, in the control group own name/face processing resulted in the highest amplitudes of P300 in comparison to all other names/faces (i.e., close-other's, famous, unknown name/face). In the ASD group, P300 for own name/face did not differ from P300 for close-other name/face. However, P300 to own and close-other's name/face was significantly higher than P300 to other (famous and unknown) name/face.

Our P300 results showing preferential processing of the self-related stimuli in the control group are in line with previous ERP studies in healthy subjects [32], [34], [36], [68], [72], [73]. The novel finding in the control group is that amplitudes of P300 to own name and face were also higher than P300 to close-other's name and face. To our knowledge, none of the previous ERP studies used such stimuli together with self, famous, and unknown names and faces. Enhanced P300 in a own face condition in comparison to a friend's face condition was reported in one study only [12].

In contrast to the control group, late P300 findings in the ASD group revealed that the self-preference effect was present only when own name/face was compared to distant others' names/faces and absent when close-other's name/face was used as a reference to the self-related stimuli. The latter suggests equivalent attention allocation for own and close-other's faces and names in the ASD group. Thus, at the level of detection, the self-related stimuli are not differentiated from the close-other related stimuli, but they are differentiated from stimuli related to the more distant other (i.e. a famous person and the unknown person). It seems that in the case of ASD individuals, preferential processing was not restricted only to the self-related stimuli but to all personally relevant stimuli. Enhanced P300 to both own and close-other's name and face in the ASD group may reflect not only similar attentional characteristics [61] of these stimuli but also emotional ones [63]. Attention and emotion may complement each other as the model of motivated attention [101] states that emotional cues prompt motivational regulation and draw attentional resources. This is supported by findings of behavioral [100] and electrophysiological studies [102]. Although lower impact of emotion in guiding attention to socially-relevant stimuli might be expected in ASD [103], it is plausible that higher P300 amplitudes to the self and close-other related stimuli in the ASD group reflect similar emotionally motivated attentional load of these stimuli. One may speculate that in the case of ASD participants, motivated attention allocation to those stimuli might be associated with a kind of behavioral learning. This supposition is supported by the fact that most participants from our ASD group (22 out of 23) chose a family member as the ‘close other’. Taking into account that they spent most of the time at home, extensive contact with that person and his/her significance in fulfilling daily needs may result in intensive stimulus- reward learning [104].

Alternative interpretations of P300 findings, not referring to the attentional processes, are also plausible. For example, our P300 findings may be interpreted in the context of the person recognition model [105]–[108] and its ERP adaptations [73], [92], [109], [110]. P300 is considered to reflect activation of semantic knowledge about a person [110]. Thus, P300 findings in the ASD group may suggest similar levels of person-specific semantic knowledge, referring to the self and the significant other. In contrast, the control group mainly displayed activation of self-knowledge. This result may support the theoretical view of a poorly developed or even absent ‘I-concept’ [22]. This is related to a distorted perception of oneself as unique and distinct from others. As a result, we can observe insufficient elaboration of the self-concept and impaired differentiation of the self from the significant other in autistic individuals [10], [22].

The P300 findings in the ASD group may also be viewed in the light of Uddin's [37] hypothesis, stating that psychological but not physical aspects of the self are altered in ASD. In other words, it might be expected that processing of ‘symbolic’ self-related stimuli (e.g. own name) is more impaired in ASD patients than processing of ‘physical’ self-related stimuli (e.g. own face). However, investigating these two types of stimuli at the same time, using the same experimental procedure, the same modality of stimuli, and with the same participants, we observed an analogous pattern of late P300 amplitudes for own name and own face. Thus our P300 results indicate that the ‘physical self’ and ‘non-physical self’ are processed in a similar way not only in the control group but also in the ASD group. Although our findings seems to disprove Uddin's hypothesis one may speculate that some in-depth processing, absent in our detection task, would be required to reveal disturbances in the ‘psychological self’. The only difference between detection of names and faces (including one's own name and one's own face) observed in both groups was related to the temporal delay of the former in comparison to the later. P300 amplitude differentiated categories of faces in the early time window and categories of names in the late time window. This may be linked to the time consuming semantic processing of name stimuli.

ERP findings of this study also reveal attenuated lateralization of face and name processing in ASD. In the control group only, name detection in general was associated with higher activity in the left hemisphere whereas face detection was associated with enhanced activity in the right hemisphere, as revealed by N170 and P300 amplitudes, respectively. In contrast, lateral differences were absent in the ASD group. All of these effects support the notion of atypical functional brain organization [111], [112] in ASD participants during social stimuli processing.

Finally, one may hypothesize that aurally presented names should bring more ecologically valid findings. The auditory version of a name is more adequate in the context of communication and social interactions. However, we used the visual version of names in order to investigate self-related stimuli that differed only in respect to their domain (‘physical’, i.e., face vs. ‘non-physical’, i.e. name), but not their modality. Results from our own neuroimaging study [42] on healthy participants suggest that the involvement of the medial prefrontal cortex, is largely independent from the modality of one's own name. However, in some other brain regions (e.g. inferior frontal gyri) the preference in processing of one's own name vs. the close-other's name was present only for the auditory modality [42]. Therefore, it cannot be ruled out that using auditory presentations of the names would reveal a different pattern of results.

In conclusion, the present study provides evidence indicating equivalent engagement of attentional resources in detection of visually presented stimuli related to the self and to the close-other in adolescent and young adults with ASD. Similar effects were observed for names and for faces. In contrast, preferential attention allocation for the self-face and self-name was observed in typically developing individuals. Further research with different tasks and stimuli is needed to fully explain the impaired ‘me’ vs. ‘not-me’ distinction in autism.
